# Supplementary material for: Effects of Dietary Ferulic Acid Supplementation on Hepatic Injuries in Tianfu Broilers Challenged with Lipopolysaccharide
Source: Toxins (Basel). 2022 Mar 21;14(3):227. doi: 10.3390/toxins14030227 (PMC8955363; doi:10.3390/toxins14030227)
Supplement: Supplementary file 1 [file toxins-14-00227-s001.zip › toxins-1633939-supplementary.pdf]

toxins

Supplementary Materials: Effects of Dietary Ferulic Acid Supplementation on Hepatic Injuries in Tianfu Broilers Challenged with Lipopolysaccharide

Gang Shu, Ziting Tang, Hong Du, Yilei Zheng, Lijen Chang, Haohuan Li, Funeng Xu, Hualin Fu, Wei Zhang and Juchun Lin

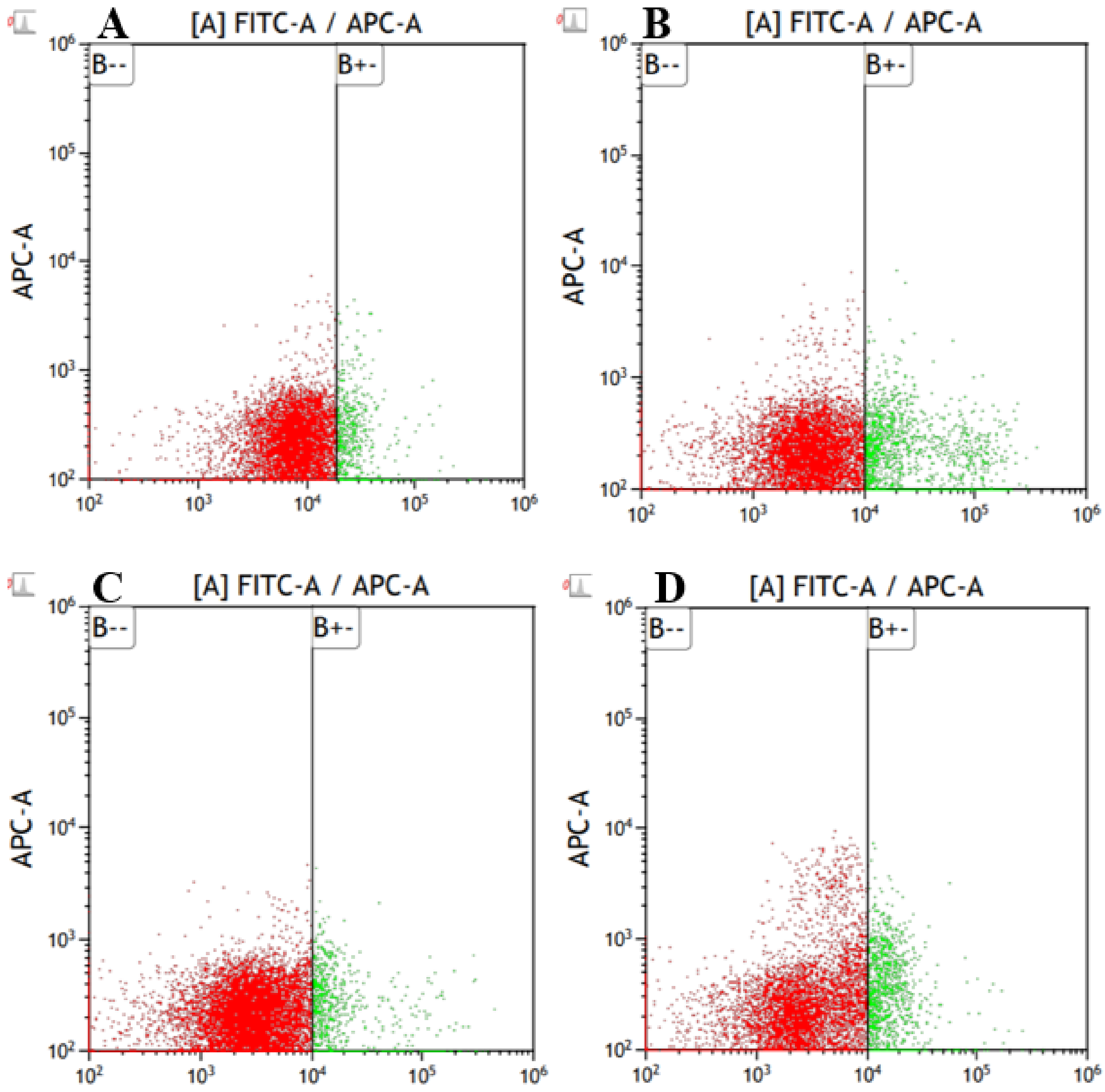

Figure S1. The flow cytometry quadrant diagrams of ROS in different groups. **A**: CON group, **B**: LPS group, **C**: FA group, **D** FL group.

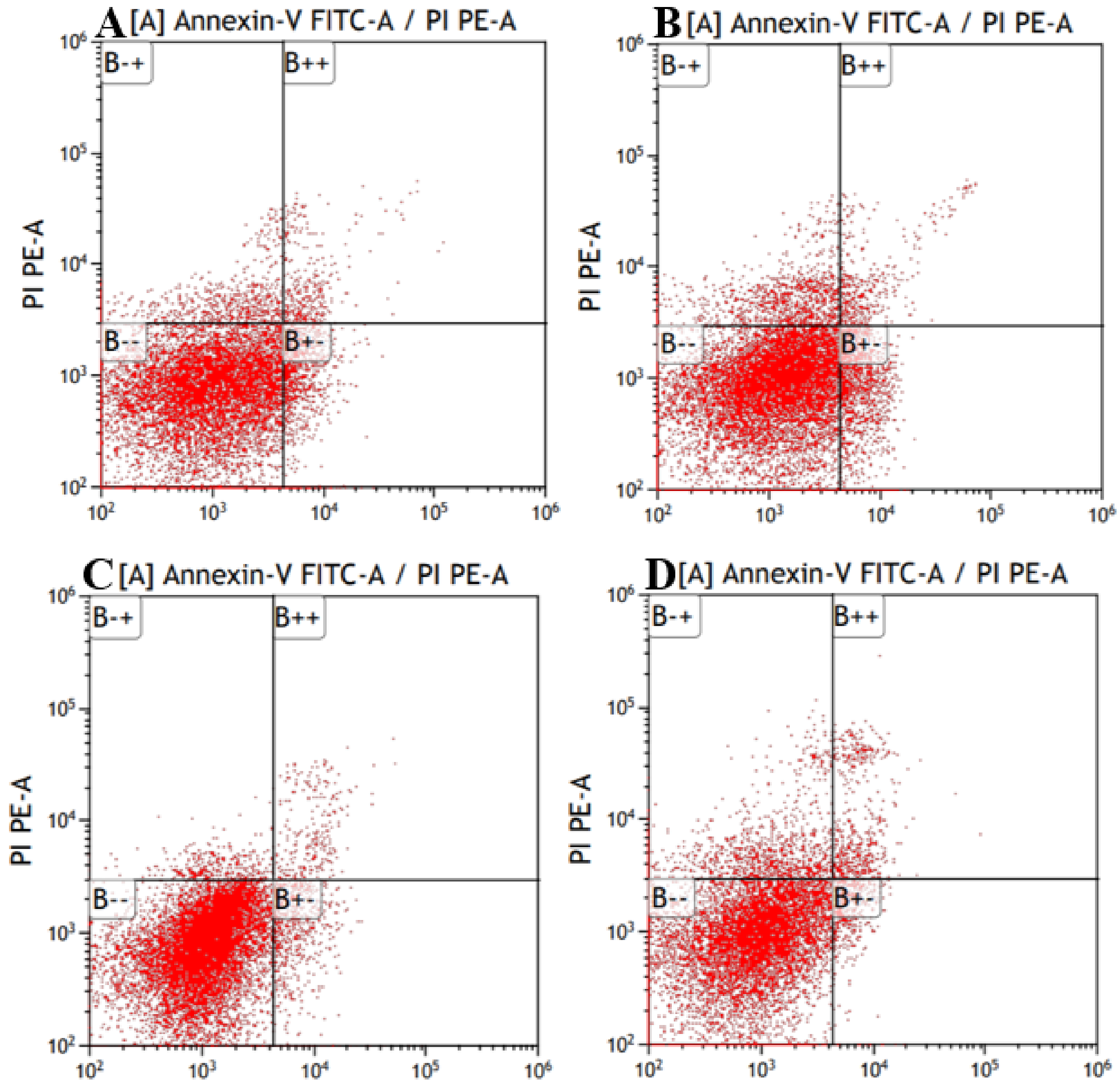

Figure S2. The flow cytometry quadrant diagrams of apoptosis in different groups. **A**: CON group, **B**: LPS group, **C**: FA group, **D** FL group.

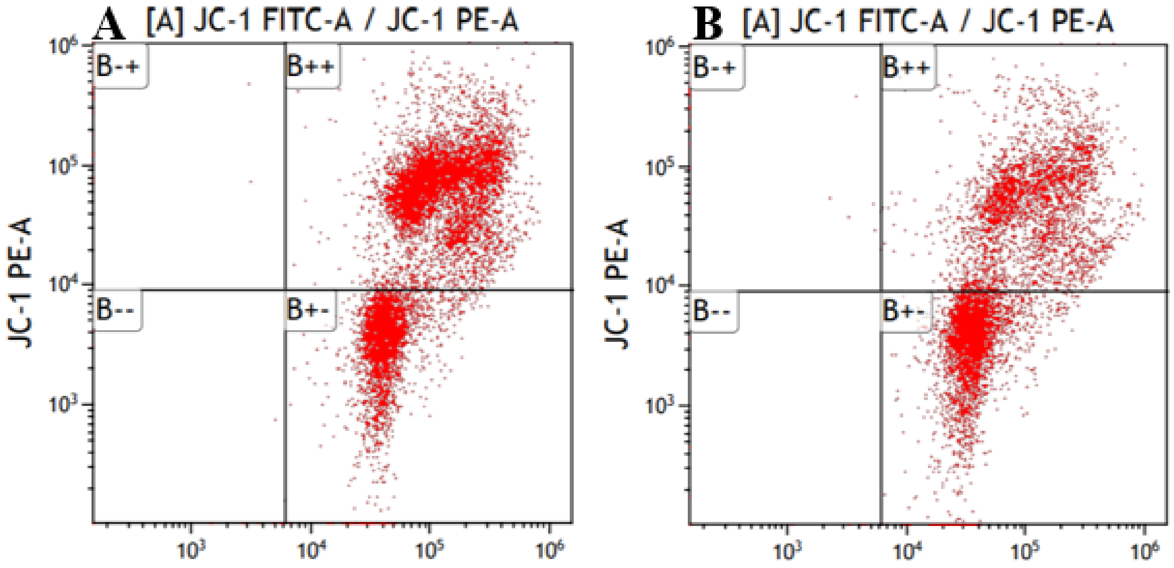

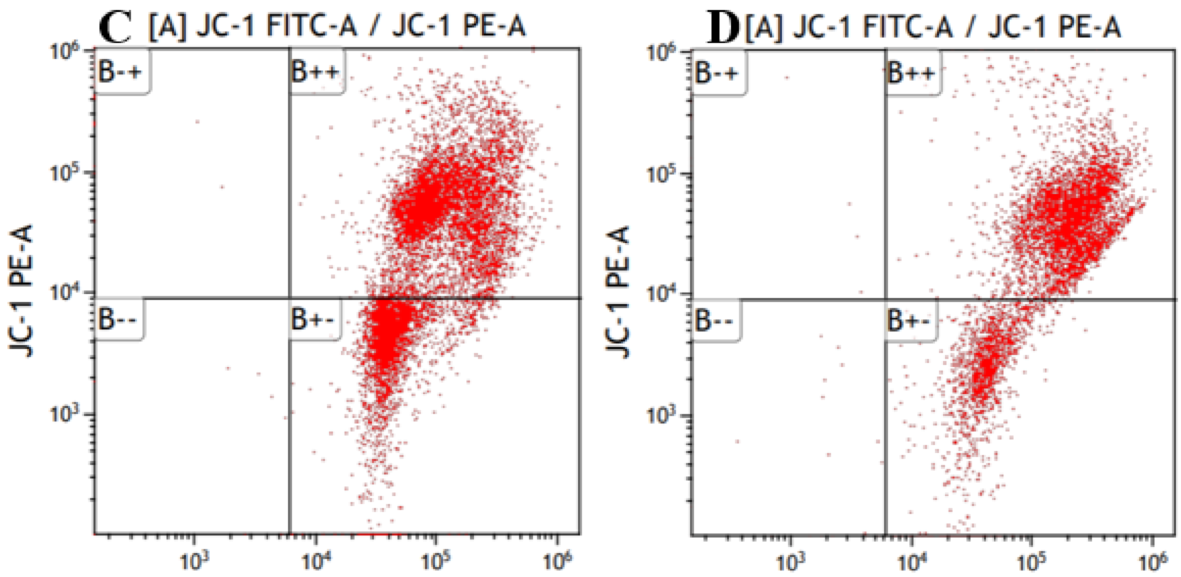

Figure S3. The flow cytometry quadrant diagrams of the Mitochondrial depolarization ratio levels in different groups. **A**: CON group, **B**: LPS group, **C**: FA group, **D** FL group.
